# Effect of Betanin, the Major Pigment of Red Beetroot (*Beta vulgaris* L.), on the Activity of Recombinant Human Cytochrome P450 Enzymes

**DOI:** 10.3390/ph16091224

**Published:** 2023-08-30

**Authors:** Sung Ho Lim, Seoungpyo Bae, Ho Seon Lee, Hyo-Kyung Han, Chang-Ik Choi

**Affiliations:** 1 Integrated Research Institute for Drug Development, College of Pharmacy, Dongguk University-Seoul, Goyang 10326, Republic of Korea; 93sho617@naver.com (S.H.L.); dgucpt001@gmail.com (S.B.); ghtjsrhtn@naver.com (H.S.L.); 2 BK21 FOUR Team and Integrated Research Institute for Drug Development, College of Pharmacy, Dongguk University-Seoul, Goyang 10326, Republic of Korea; hkhan@dongguk.edu

**Keywords:** *Beta vulgaris* L., betanin, cytochrome P450 enzymes, fluorescence-based assay, HepG2 cells, herb–drug interactions, luminescence-based assay

## Abstract

Most of the currently available drugs are derived from natural sources, but they are used only after extensive chemical modifications to improve their safety and efficacy. Natural products are used in health supplements and cosmetic preparations and have been used as auxiliary drugs or alternative medicines. When used in combination with conventional drugs, these herbal products are known to alter their pharmacokinetics and pharmacodynamics, reducing their therapeutic effects. Moreover, herb–drug interactions (HDIs) may have serious side effects, which is one of the major concerns in health practice. It is postulated that HDIs affect the pathways regulating cytochrome P450 enzymes (CYPs). Betanin, the chief pigment of red beetroot (*Beta vulgaris* L.), has various types of pharmacological activity, such as anti-inflammatory, antioxidant, and anticancer effects. However, the potential risk of HDIs for betanin has not yet been studied. Thus, we aimed to predict more specific HDIs by evaluating the effects of betanin on CYPs (CYP1A2, CYP2B6, CYP2C9, CYP2C19, CYP2D6, and CYP3A4), the major phase I metabolic enzymes, using fluorescence-/luminescence-based assays. Our results showed that betanin inhibited CYP3A4 activity in a dose-dependent manner (IC_50_ = 20.97 µΜ). Moreover, betanin acted as a competitive inhibitor of CYP3A4, as confirmed by evaluating Lineweaver–Burk plots (*Ki* value = 19.48 µΜ). However, no significant inhibitory effects were observed on other CYPs. Furthermore, betanin had no significant effect on CYP1A2, CYP2B6, or CYP2C9 induction in HepG2 cells. In conclusion, betanin acted as a competitive inhibitor of CYP3A4, and thus it should be used cautiously with other drugs that require metabolic enzymes as substrates. Additional in vivo studies and clinical trials are needed to further elucidate the HDIs of betanin.

## 1. Introduction

Traditional medicines, such as Ayurveda, Unani, traditional Chinese medicine, Kampo, and traditional Korean medicine, use natural products and have been part of a worldwide organized and regulated medical system for hundreds or thousands of years [1]. Traditional medicines are used to treat various diseases [2], and several major drugs used in clinical settings are derived from natural sources, such as plants, animals, and microorganisms [3].

In particular, it has been widely documented that approximately 80% of the world’s population has traditionally relied on plants for the prevention and treatment of various diseases [4]. Herbal medicines are a promising option over modern synthetic drugs because of their lower cost and fewer side effects, and they are considered safe and effective in treating human diseases [5]. In addition, many plant constituents and numerous chemical compounds with different biological and pharmacological activity have been isolated from medicinal plants [6].

For instance, clinical trials have shown that natural compounds prevent radiation- induced bladder and intestinal dysfunction as well as erectile dysfunction [7]. Phytochemicals may also be used as promising alternatives to current therapies for neurodegenerative disorders owing to their anti-inflammatory, antioxidant, and anticholinesterase activity [8]. In addition, several natural products with reactive oxygen species (ROS) scavenging activity protect against oxidative damage, acute damage from aging, exposure to radiation and chemicals, and long-term toxicity risks [9]. Natural products with antihyperglycemic effects can be used as a potentially beneficial adjuvant therapy with antidiabetic drugs [10].

Natural products are usually administered as an adjuvant or as an alternative medicine with conventional drugs [11]. However, the growing popularity of alternative medicines worldwide raises new concerns about herb–drug interactions (HDIs), which can lead to serious side effects and decreased pharmacological efficacy [12]. Several drug metabolism studies have been conducted to efficiently predict the side effects caused by HDIs [13]. An important mechanism closely related to HDIs is the regulation of cytochrome P450 enzymes (CYPs), the major phase I metabolic enzymes [14]. The CYP superfamily comprises CYP1A2 (%), CYP2B6 (3%), CYP2C9 (16%), CYP2C19 (8%), CYP2D6 (19%), CYP3A4 (36%), and other CYPs (7%) [15]. In particular, the CYP1, 2, and 3 families are involved in the metabolism of 70–80% of all drugs [16]. Various herbs and natural compounds have the potential to affect CYP expression and may interfere with drug metabolism [17]. For instance, a clinical trial showed that green tea (*Camellia sinensis*) weakly inhibits CYP3A4, an enzyme that metabolizes simvastatin [18]. By stopping the co-administration of green tea and simvastatin, tolerance to the drug can be improved [19]. In addition, quercetin and tangeretin, compounds that possess anti-inflammatory, antidiabetic, and antioxidant properties, upregulate the expression of CYP3A4 and have inhibitory effects on P-glycoprotein (P-gp)-mediated drug metabolism [20,21].

Red beetroot (*Beta vulgaris* L.) is a valuable natural pigment source that is inexpensive and easy to sustain and is also attracting attention as a ‘superfood’, a nutrient-rich natural food [22]. It belongs to the botanical order *Chenopodiaceae* [23]. Red beetroot is a Middle Eastern species that has spread worldwide from the Americas to Europe and Asia, where it is grown commercially as an annual or biennial [24]. Red beetroot extract has shown potential for use in diseases associated with chronic inflammation, liver disease, arthritis, and even cancer through its antioxidant and anti-inflammatory properties in in vitro studies and in vivo animal models [25]. It contains various bioactive compounds such as betalains, betaxanthins, and betacyanins and has long been used as a medicinal component and food supplement [26]. Betanin, a water-soluble red pigment that accounts for 75–95% of the total betalain content [27], possesses various types of pharmacological activity, such as anti-inflammatory, antioxidant, and anticancer activity [28,29,30]. Pharmacokinetic studies of betanin have advanced considerably, encouraging further in vivo studies using the pure molecule to investigate betanin’s potential bioactivity [31,32]. The use of red beetroot extract, known for its anticancer activity, in an appropriate combination and dose can improve the therapeutic efficacy of chemotherapy drugs such as doxorubicin and reduce toxic side effects through dose reduction [33]. However, HDIs for betanin have not yet been investigated.

Traditional methods of examining CYP activity are centered on chromatographic analysis [34]. Fluorescence-based assays are a new and valuable tool for the study of CYPs’ metabolism and inhibition in humans and for the screening of multiple drugs and compounds for CYP isozyme-specific inhibitors and HDIs [35]. Liquid chromatography–mass spectrometry-based assays allow the detection of the levels of metabolites of interest but require large amounts of enzymes and are difficult to perform. In contrast, fluorescence-/luminescence-based assays minimize the potential solvent effects by reducing the proportion of organic solvent present in the reaction. These assays are a robust and reproducible tool for the rapid investigation of the interactions of natural compounds with P450 family enzymes [36,37]. Moreover, fluorescence-/luminescence-based assays allow the accurate detection of isoform-specific inhibition by nanomolar concentrations of potent CYP inhibitors and the discrimination of different substrates of various CYP isoforms [38]. Therefore, this study aimed to predict more specific HDIs by evaluating the effect of betanin on CYP modulation using recombinant CYPs and HepG2 cells, by employing a fluorescence-/luminescence-based assay.

## 2. Results

### 2.1. Inhibitory Effect of Betanin on CYP Activity Using Fluorescence-Based Assay

**Figure 1:**
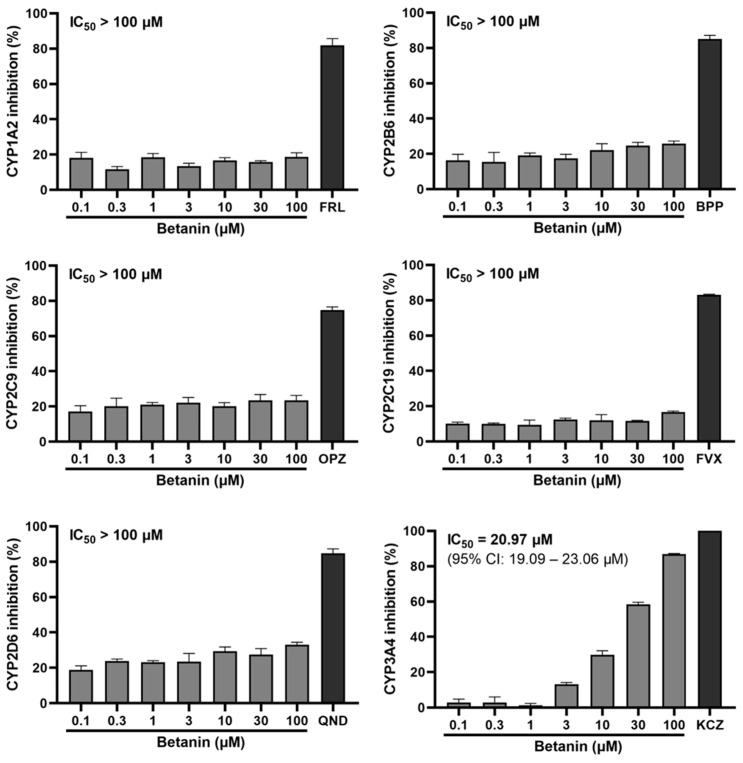
Inhibitory effect of betanin on recombinant CYPs evaluated using commercial Vivid CYP450 screening kits. Raw fluorescence data were normalized against control activity values of 0% and 100%. Control 0% activity values were obtained from samples consisting of 0.5% DMSO and the enzyme solvent buffer without the enzyme. Maximum 100% activity values were expressed as a reaction with only enzymes and 0.5% DMSO, with no other drugs. Furafylline (FRL) 100 μM, bupropion (BPP) 50 μM, omeprazole (OPZ) 50 μM, fluvoxamine (FVX) 100 μM, quinidine (QND) 50 μM, and ketoconazole (KCZ) 1 μM were used as positive controls for the fluorescence-based assay. Betanin/positive inhibitor dilutions all contained 0.5% DMSO to prevent results from being distorted by variations in DMSO levels. Raw data were normalized using the software GraphPad Prism 8.02. The data represent the mean ± SEM of triplicate independent experiments.

We measured the activity of six representative CYPs using Vivid CYP screening kits. CYP activity was evaluated using recombinant CYPs and specific fluorescent substrates 7-ethoxymethoxy-3-cyanocoumarin (EOMCC) and 7-benzyloxymethyloxy-3-cyanocoumarin (BOMCC). The potent CYP inhibitors furafylline (for CYP1A2), bupropion (for CYP2B6), omeprazole (for CYP2C9), fluvoxamine (for CYP2C19), quinidine (for CYP2D6), and ketoconazole (for CYP3A4) were used. As shown in Figure 1, the activity of recombinant CYP1A2, CYP2B6, CYP2C9, and CYP2C19 was not modulated by betanin, and its inhibitory effect was not above 30% at any of the tested dosages. In contrast, betanin (100 μM) showed a 33% weaker inhibitory effect on recombinant CYP2D6, being below that of quinidine, even at the highest concentration. However, betanin (0.1–100 μM) showed a significant inhibitory effect on CYP3A4 in a dose-dependent manner. In particular, 30 and 100 μM betanin showed strong inhibition by 58% and 87%, respectively. The IC_50_ value for the inhibition of recombinant CYP3A4 by betanin was 20.97 μM.

### 2.2. Inhibitory Mechanism of Betanin on CYP Enzyme Activity

**Figure 2:**
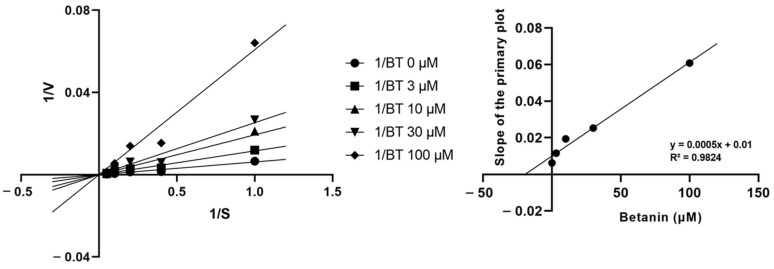
Lineweaver–Burk plot and the secondary plot for *Ki* of inhibition of betanin on CYP3A4. Data were obtained from 30 min incubation with BOMCC (1–20 μM) in the absence or presence of betanin (3–100 μM). All data are represented as mean ± SD from at least three independent experiments.

Next, we measured the kinetics of betanin metabolization by recombinant CYP3A4. Using the Michaelis constant (Km) and Vmax, we generated a Lineweaver–Burk plot. To obtain inhibition constant (*Ki*) values, various concentrations of BOMCC were incubated in the absence or presence of 3–100 μM betanin. CYP3A4 was most effectively inhibited in a competitive manner, and the *Ki* was 19.48 μM after incubating 1–20 μM BOMCC in the presence of 3, 10, 30, and 100 μM betanin (Figure 2).

### 2.3. Induction Effect of Betanin on CYP Activity Using Luminescence-Based Assay

**Figure 3:**
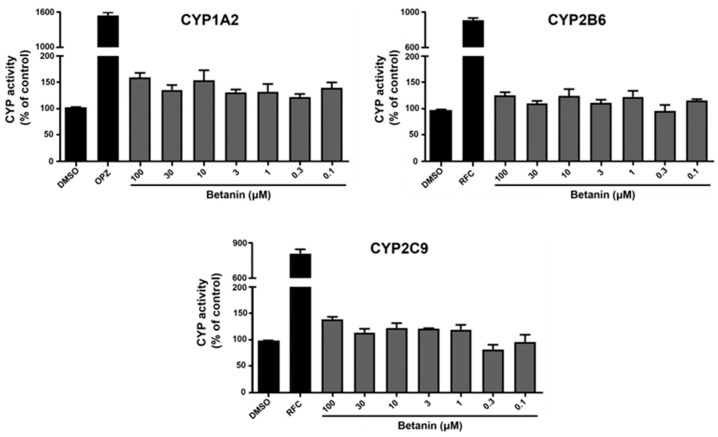
Induction effect of betanin on CYP1A2, CYP2B6, and CYP2C9. CYP enzyme activity was measured using P50-Glo assay kits. Omeprazole (OPZ) 1 mM and rifampicin (RFC) 1 and 10 mM were used as positive controls for the luminescence-based assay. The data are expressed as a percentage of the control (DMSO 0.5%). Results are mean ± SEM of triplicate independent experiments.

To determine the effect of betanin on HepG2 cell viability, we performed MTT assays. HepG2 cells were cultured for 24 h and then incubated with betanin (0.1–100 μM) for another 48 h. Within the tested range, betanin showed no cytotoxicity toward HepG2 cells. Based on these results, we used P450-Glo assays to determine the effects of betanin on the activity of three representative CYPs (CYP1A2, CYP2B6, and CYP2C9) by measuring the release of luciferins (Luciferin-1A2, Luciferin-2B6, and Luciferin-H). HepG2 cells (70% confluence) were treated with the inducers 1 mM omeprazole (for CYP1A2) or 10 mM rifampin (for CYP2B6 and CYP2C9). As shown in Figure 3, treatment with each inducer significantly induced CYP2A2, CYP2B6, and 2CYP3C9 compared to the control group, whereas betanin did not show any induction effect at the concentration tested.

## 3. Discussion

Herbal compounds are often administered with conventional drugs for the treatment of major diseases, although the likelihood of HDIs is high [39]. Plant-derived natural compounds exert therapeutic effects because they possess antioxidant and anti-inflammatory properties, among others [40]. HDIs affect the pharmacokinetics and pharmacological properties of drugs by altering the activity of drug-metabolizing enzymes and transport proteins such as CYPs and P-gp [21,41]. Therefore, the potential interactions of herbal products with prescription or non-prescription medicines are the focus of global research efforts for the successful treatment of diseases [42].

The increased interest in so-called “functional foods” and their applications in health and disease is a result of the health advantages of diets rich in fruits and vegetables. *B. vulgaris*, commonly known as red beetroot, has recently gained popularity as a health-promoting functional food [43]. Betanin, one of the components of beetroot, has several beneficial physiological effects [44]. Betanin has attracted attention for its anti-inflammatory, antioxidative, and hepato-protective effects on human hepatocytes [45]. Betanin also improves hypotension, lowers total plasma cholesterol and triglyceride levels, and improves cardiovascular structure and function [29].

Betanin is metabolized by the major phase I enzymes, CYPs. Among CYPs, the CYP1, 2, and 3 families are associated with 70–80% of the metabolism of all clinically used drugs. In particular, CYP3A4 metabolizes more than half of the most commonly used drugs [46]. Thus, numerous therapeutic compounds, including those from natural products, are substrates of CYP3A4 [47]. Betanin suppresses CYP3A2 mRNA expression and CYP2E1 activity [48,49], but its effects on other CYPs have not yet been reported. Therefore, we evaluated the effect of betanin on six major drug-metabolizing CYPs to identify its potential HDIs.

Herein, we used a fluorescence-based assay with Vivid CYP assay substrates (BOMCC and EOMCC) containing coumarin, NADP+, and a fluorescent substrate based on the NADPH regeneration system. This assay has several advantages. First, the use of a fluorogenic substrate with CYP enzymes allows the rapid and robust detection of the interactions between test compounds and the P450 family enzymes. Second, this method has better precision and sensitivity compared to mass spectrometry-based assays. Moreover, it is economical and easy to conduct [36]. Betanin significantly and dose-dependently inhibited the activity of recombinant CYP3A4, which is similar to the effect of ketoconazole, a potent CYP3A4 inhibitor. However, it had a weak inhibitory effect on CYP2D6 and no significant inhibition by betanin was observed for other CYPs (CYP1A2, CYP2B6, CYP2C9, and CYP2C19).

Next, we evaluated the enzyme kinetics of CYP3A4 inhibition by betanin. Competitive, non-competitive, and uncompetitive inhibition modes are the three main classifications for the reversible inhibition of enzyme activity. Competitive inhibition occurs when the inhibitor and substrate compete for the same active site, and an increase in the substrate concentration can overcome competitive inhibition because the competitive inhibitor and structural analogue of the substrate compete for the same binding site. In non-competitive inhibition, the inhibitor binds to both the enzyme and the enzyme–substrate complex. In uncompetitive inhibition, the enzyme–substrate complex serves as the binding site for the inhibitor, which increases the availability of substrates by stopping the reaction between the enzyme and the substrate [50]. In the present study, the Lineweaver–Burk and secondary plots showed that betanin operated in competitive mode. These results suggest that the betanin-mediated inhibition of CYP3A4 may have adverse consequences in treatments with drugs metabolized by CYP3A4.

Subsequently, we used a luminescence-based assay with HepG2 cells to evaluate the potential induction effects of betanin on CYP1A2, CYP2B6, and CYP2C19. The induction of CYPs in HepG2 cells is closely related to the CYP induction observed in human primary hepatocytes [51]. The P450-Glo assay employed in this study uses luminescent CYP substrates that are derivatives of beetle luciferin, a substrate for the luciferase enzyme. The derivative, which is not a substrate for luciferase, is converted into luciferin by CYPs. The P450-Glo substrates and the reaction products of this assay are cell-permeable. This allows intracellular CYPs to convert substrates into luciferin products, which can be detected using the luciferin detection reagent after exiting HepG2 cells. Thus, luminescence is proportional to CYP activity. These luminescence assays exhibit excellent sensitivity, a low background signal, and a wide dynamic range. The combined use of fluorescence- and luminescence-based assays provides a reliable preclinical platform for the detection of the effects (inhibition and induction) of test compounds on CYPs [47]. Our results showed that betanin had no significant induction effects on CYP1A2, CYP2B6, or CYP2C9 in HepG2 cells.

Taken together, our results suggest that betanin acts as a competitive inhibitor of CYP3A4, and thus caution is advised when using it in combination with other drugs that are substrates of these enzymes. However, the factors affecting HDIs are diverse, including genetics, age, gender, disease state, social factors, and diet. Further validation and in vivo studies are needed to establish the clinical significance of the observed HDI potential. In addition, the results need to be replicated and validated using other methods or models at the in vitro level. For example, drug metabolism can be achieved through non-CYPs such as uridine diphosphate glycosyl-transferases and sulfotransferases, which are phase II hepatic metabolic enzymes. Furthermore, transporters such as P-gp and multi-drug resistance 1 protein can have clinically significant effects on the pharmacokinetics and pharmacodynamics of drugs in various organs and tissues by controlling the absorption, distribution, and elimination of drugs [52]. The lack of these experiments is a limitation of our study, and further studies will enable us to reveal the clinical relevance of betanin for HDIs.

## 4. Materials and Methods

### 4.1. Reagents and Recombinant CYPs

Dimethyl sulfoxide (DMSO) was provided by Glentham Life Sciences (Corsham, UK). PCR-grade nano-pure water was purchased from Jena Bioscience (Munich, Germany). Vivid CYP1A2, CYP2B6, CYP2C19, CYP2D6, and CYP3A4 screening kits, EOMCC, and BOMCC were purchased from Thermo Fisher Scientific (Waltham, MA, USA). Betanin, bupropion, furafylline, omeprazole, quinidine, rifampicin, and Krebs-Henseleit buffer were supplied by Sigma-Aldrich (St. Louis, MO, USA). Sulfaphenazole, fluvoxamine, and ketoconazole were obtained from Toronto Research Chemicals (Toronto, ON, Canada). P450-Glo CYP1A2, P450-Glo CYP2B6, and P450-Glo CYP2C9 assay kits were provided by Promega (Wisconsin, MD, USA).

### 4.2. Fluorescence-Based CYP Inhibition Assay

Fluorescence-based assays were performed according to the manufacturer’s instructions. Control, CYP positive control inhibitor, and betanin (0.1–100 μM) solutions were prepared by diluting the compounds with Vivid CYP Reaction Buffer (Table 1). Next, 40 µL of control, CYP positive control inhibitor, or betanin solution was dispensed into the wells of black 96-well plates. A Master Pre-Mix was prepared by diluting the P450 BAC-ULOSOMES Plus Reagent and Vivid Regeneration System in Vivid CYP Reaction Buffer. The P450 BACULOSOMES Plus Reagents are microsomes made from insect cells that express human cytochrome P450 reductase and CYP450 isozyme. After 15 min of pre-incubation, 10 µL of a mixture of Vivid Substrate and NADP+ was pipetted into the wells of a black 96-well plate to start the enzymatic reaction. The plates were immediately measured in the kinetic mode using a SpectraMax M3 Multi-Mode Microplate Reader (Bio-Rad, Hercules, CA, USA). The excitation and emission wavelengths for each Vivid recombinant CYP are shown in Table 2. IC_50_ determination for human CYPs was performed as described by Crespi et al. [53]. Incubation was implemented with 1% acetonitrile in a total volume of 100 μL potassium phosphate buffer (50 mM, pH 7.4). A reaction mixture containing the NADPH regeneration system in potassium phosphate buffer (pH 7.4) and various inhibitors (dissolved in 100% acetonitrile) was pre-incubated at 37 °C for 10 min (excluding CYP3A4, which was dissolved in 100 mM Tris buffer, pH 7.4). The above-mentioned solution was reacted with betanin and the enzyme substrate at 37 °C for 30 min. The signals were measured using a SpectraMax M3 Multi-Mode Microplate Reader (Bio-Rad). Data fitting generated a sigmoidal dose–response curve with values calculated using Microsoft Excel 2016 (Microsoft Corporation, Redmond, WA, USA).

### 4.3. Determination of CYP Activity

CYP activity was measured using Vivid CYP screening kits supplied with P450 BACULOSOMES Plus Reagent containing each CYP. The assays were conducted in the absence or presence of betanin and different concentrations of specific substrates. BOMCC was used as a specific substrate for CYP3A4. Lineweaver–Burk plots were generated for the determination of CYP activity in the absence or presence of betanin.

### 4.4. HepG2 Cell Culture and Viability Assay

Human hepatocellular carcinoma (HepG2) cells were cultured in Minimum Essential Medium (MEM) supplemented with 10% fetal bovine serum and 1% penicillin–streptomycin. HepG2 cells were maintained at 37 °C in a humidified environment with a 5% CO₂ atmosphere. The culture medium was replaced every day before measuring CYP activity. HepG2 cells were grown in opaque 96-well plates at a density of 1 × 10^4^ cells/well. After 24 h, the cells were added with the desired concentration of betanin for 48 h. Next, 20 µL of 5 mg/mL MTT solution in PBS was added to each well and incubated for 2 h. After removing the supernatant, 100 µL of DMSO was dispensed into each well and the plate was incubated for 10 min. The absorbance was measured at 540 nm using a SpectraMax M3 Multi-Mode Microplate Reader (Bio-Rad).

### 4.5. Luminescence-Based CYP Induction Assay

HepG2 cells were seeded at a concentration of 3 × 10^4^ cells per well for CYP1A2 and 1 × 10^5^ cells per well for CYP2B6 and CYP2C9 in opaque white 96-well plates. After 24 h of incubation, the cells were combined with 0.5% DMSO (vehicle control), 1 mM omeprazole, 10 mM rifampicin, or 0.1–100 μM betanin for 48 h. The supernatant was then switched to fresh medium containing the same concentration of the compound and incubated for an additional 24 h. To measure luminescence, the culture medium was replaced with fresh medium containing the luciferin substrate and incubated at 37 °C in a 5% CO₂ incubator (Table 2). Next, 50 µL of each well was transferred to a well of a fresh 96-well opaque white cell culture plate and mixed with 50 µL of CYP Luciferin Detection Reagent at room temperature to initiate the enzymatic reaction. After incubation for 20 min, luminescence was measured using a SpectraMax M3 Multi-Mode Microplate Reader (Bio-Rad).

### 4.6. Statistical Analysis

All measurements were performed in kinetic mode using the assay and instrument settings described above. The results were compared to those of the control reactions performed in the absence of enzyme components. Quantitative data are represented as the mean ± standard deviation (SD) from at least three independent experiments. All statistical analyses were performed with GraphPad Prism 8 (GraphPad Software, San Diego, CA, USA). Differences between the control and treatment groups were investigated using the Student *t*-test.

## 5. Conclusions

In summary, this study suggests for the first time that the ingestion of betanin-containing foods or medicines along with drugs metabolized by CYP3A4 can induce HDIs, reducing the therapeutic efficacy of the drug or leading to drug toxicity. This in vitro study reveals the potential HDI mechanism of betanin that may affect pharmacokinetics, and it provides mechanistic information necessary for clinical evaluation and study design together with clinical pharmacokinetic data. Therefore, further evaluation based on clinical pharmacokinetic data is needed, and in vivo HDI investigations should be conducted if necessary. We believe that our study provides useful insights into the nature of CYP inhibition by betanin.

## Figures and Tables

**Table 1:** Experimental conditions for the fluorescence-based assay with recombinant CYP enzymes.

P450 Isoform	Substrate	Positive Inhibitor (µM)	Incubation Time (min)	Wavelengths (nm)	
				Excitation	Emission
CYP1A2	EOMCC	Furafylline, 5	15	408	455
CYP2B6	BOMCC	Bupropion, 50	30	409	460
CYP2C9	BOMCC	Omeprazole, 50	45	408	455
CYP2C19	EOMCC	Fluvoxamine, 100	30	408	455
CYP2D6	EOMCC	Quinidine, 50	15	405	450
CYP3A4	BOMCC	Ketoconazole, 1	10	409	460

BOMCC, 7-benzyloxymethyloxy-3-cyanocoumarin; EOMCC, 7-ethoxymethoxy-3-cyanocoumarin.

**Table 2:** Experimental conditions for the luminescence-based assay with HepG2 cells.

HepG2 Cell	Luminogenic Substrate	Substrate Concentration (µM)	Incubation Time (min)
CYP1A2	Luciferin-1A2	6	120
CYP2B6	Luciferin-2B6	3	150
CYP2C9	Luciferin-H	100	150

## Data Availability

The data presented in this study are available on request from the corresponding author.

## Abbreviations

BOMCC	7-benzyloxymethyloxy-3-cyanocoumarin
BPP	bupropion
CYPs	cytochrome P450 enzymes
DMSO	dimethyl sulfoxide
EOMCC	7-ethoxymethoxy-3-cyanocoumarin
FRL	furafylline
FVX	fluvoxamine
HDIs	herb–drug interactions
KCZ	ketoconazole
Ki	inhibition constant
MEM	Minimum Essential Medium
OPZ	omeprazole
P-gp	P-glycoprotein
QND	quinidine
RFC	rifampicin
ROS	reactive oxygen species
